# Impact of chronic methamphetamine treatment on the atherosclerosis formation in ApoE−/− mice fed a high cholesterol diet

**DOI:** 10.18632/oncotarget.19020

**Published:** 2017-07-05

**Authors:** Pengfei Zhu, Lun Li, Bo Gao, Mingjing Zhang, Yuting Wang, Ye Gu, Liqun Hu

**Affiliations:** ^1^ Department of Cardiology, Heart Center at Puai Hospital, Puai Hospital, Huazhong University of Science and Technology, Wuhan 430030, China

**Keywords:** atherosclerosis, methamphetamine, immune balance, inflammation

## Abstract

**Background:**

We previously reported that methamphetamine could promote atherosclerosis (AS) in ApoE−/− mice fed normal chow. We herein observed the impact of methamphetamine on AS in ApoE−/− mice fed a high cholesterol diet and explored the potential mechanisms.

**Results and Materials and Methods:**

Male ApoE−/− mice fed a high cholesterol diet were treated with saline (NS, *n* = 5) or methamphetamine [8 mg/kg/day (M8, *n* = 6) through intraperitoneal injection] for 24 weeks. Afterwards, the percentage area of atheromatous plaque in aortic root (44.31 ± 3.21% vs. 32.91 ± 3.58%, *P* < 0.01) and atherosclerotic lesion area on Oil red O stained en face aorta (32.74 ± 6.97% vs. 18.72 ± 3.65%, *P* < 0.01) were significantly higher in M8 group than in NS group. The percentages of Th1 cells and Th17 cells in spleen were significantly higher while the percentages of Th2 cells and CD4^+^CD25^+^Foxp3^+^ Tregs were significantly lower in M8 group than in NS group. mRNA expressions of TNF-α, IFN-γ, and IL-17 were significantly up-regulated, IL-4, IL-10, Foxp3, and TGF-β were significantly down-regulated in carotid artery and in spleen in M8 group compared to NS group.

**Conclusions:**

Chronic methamphetamine treatment can enhance atherosclerotic plaque formation possibly through promoting proinflammatory cytokine secretions in ApoE−/− mice fed a high cholesterol diet.

## INTRODUCTION

Atherosclerosis is a chronic inflammatory disease with complex pathomechanisms. Mounting evidences indicate that immune and inflammation responses play major roles in the pathogenesis of atherosclerosis [1]. It is known that vascular endothelial injury, lymphocytes aggregation, and lipid deposition are the most important pathogenic determinants in the initiation and progression of atherosclerosis [2]. Previous studies also showed that T-cells mediated chronic inflammation response was actively involved in atherogenesis, and regulatory T cells (Treg) derived from CD4^+^ T cells, including CD4^+^CD25^+^Foxp3^+^ Tregs and CD4^+^ Latency-associated peptide (LAP)+ Tregs regulatory T cells, play atheroprotective role via suppressing the activity of proatherogenic effector T cells and secreting anti-inflammatory cytokines including IL-10, TGF-β, and IL-35 [3, 4]. Recently, Mallat et al. hypothesized that an imbalance of pro-atherogenic Th1 and anti-atherogenic Th2 cells might be responsible for promoted atherosclerosis [5]. Moreover, a potential role of Th17 cells and IL-17 in the pathogenesis of atherosclerosis was suggested by experimental studies [6, 7].

Previous studies revealed that chronic methamphetamine (METH) abuse is related to increased risk of cardiovascular disease including coronary atherosclerosis partly due to METH-induced sympathetic nerve activation [8, 9]. A clinical research demonstrated that the prevalence of pathologically verified typical coronary artery atherosclerosis was as high as 54% among METH decedents [10]. We recently showed that METH could promote inflammation and atherosclerotic plague formation in ApoE−/− mice fed normal chow and neuropeptide Y (NPY) might be involved in the pathogenesis of METH-induced atherogenic effects through NPY Y1 receptor pathway. It remains unknown if METH might have synergetic effects on enhancing atherosclerosis in ApoE−/− mice fed a high cholesterol diet. In the present study, we observed the impact of METH on atherosclerosis formation in ApoE−/− mice fed a high cholesterol diet and tested the hypothesis that METH might aggravate atherosclerosis.by inducing the immune imbalance characterized by reduced Tregs and increased Th1 cells in this animal model.

## RESULTS

### Methamphetamine aggravated dyslipidemia in ApoE−/− mice fed a high cholesterol diet

As shown in Table 1, body weight and plasma triglyceride level tended to be higher in M8 group compared to NS group; plasma total cholesterol and LDL-C values were significantly higher, while HDL-C was significantly lower in M8 group than in NS group.

**Table 1:** Body weight and plasma lipids

	NS	M8	*P* Values
BW (g)	27.5 ± 3.46	31.28 ± 4.6	0.165
Cholesterol (mg/dl)	607.15 ± 104.14	903.53 ± 124.0	0.002
Triglyceride (mg/dl)	600.09 ± 207.28	910.97 ± 292.32	0.078
LDL-C (mg/dl)	573.95 ± 91.75	879.64 ± 221.94	0.018
HDL-C (mg/dl)	108.03 ± 33.23	42.86 ± 25.31	0.005

BW, body weight; LDL-c, low-density lipoprotein; HDL-c, high-density cholesterol.

### Methamphetamine promoted the formation of atherosclerosis

**Figure 1:**
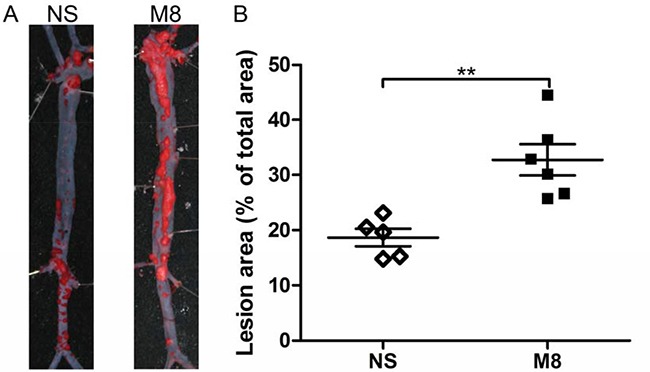
(**A**) Representative oil Red O staining of surface lesion area in the whole aorta. (**B**) Quantitative analysis of the percentage of oil Red O–positive staining area in the atheroma area. **P < 0.01.

**Figure 2:**
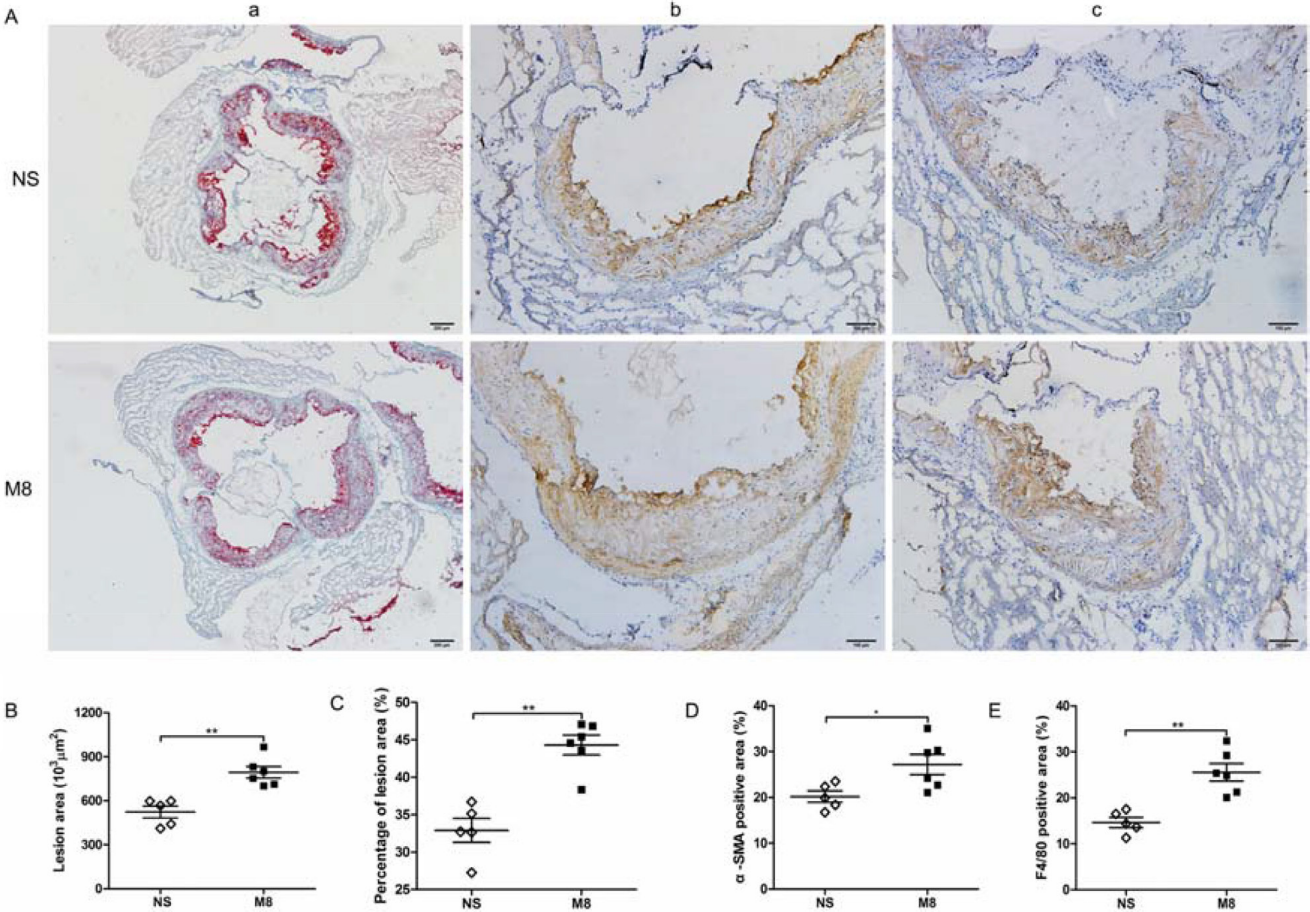
(**A**) Representative sections of aortic root stained with oil Red O (a) and specific antibodies for α-smooth muscle actin staining (b), and F4/80 for macrophages (c). Black bar in oil Red O staining represents 100 μm and in immunohistochemistry staining represents 200 μm. (**B**–**E**), Quantitative analysis of lesion size, percentage of plaque area, α-SMA, and F4/80 staining in two groups. *P < 0.05, **P < 0.01.

Results from oil Red O stained en face aorta showed that the percentage of atherosclerotic plaque was significantly increased in M8 group compared to NS group (32.74 ± 6.97% vs. 18.72 ± 3.65%, *P <* 0.01; Figure 1A and 1B). Similarly, plaque area in aortic root was significantly larger in M8 group compared to NS group (794.76.3 ± 98.29 × 10^3^ μm^2^ vs. 523.09 ± 89.89 × 10^3^ μm^2^, *P <* 0.01; Figure 2A and 2B) and percentage of atherosclerotic luminal cross sectional area (LCSA) was significantly higher in M8 group compared to NS groups (44.31 ± 3.21% vs. 32.91 ± 3.58%, *P <* 0.01; Figure 2C).

### Methamphetamine up-regulated α-SMA and F4/80 expression in aortic root plaque

Results of immunohistochemistry staining on aortic root plaque demonstrated that percent of positive stained area of α-SMA or F4/80 was significantly larger in M8 group than in NS group (27.2 ± 5.39% vs. 20.2 ± 2.77%, *P <* 0.05; 25.57 ± 4.67% vs. 14.66 ± 2.45%, *P <* 0.01; Figure 2D and 2E).

### Methamphetamine increased Th1 and Th17 cells, but suppressed Th2 and CD4^+^CD25^+^Foxp3^+^ Tregs cells in spleen

**Figure 3:**
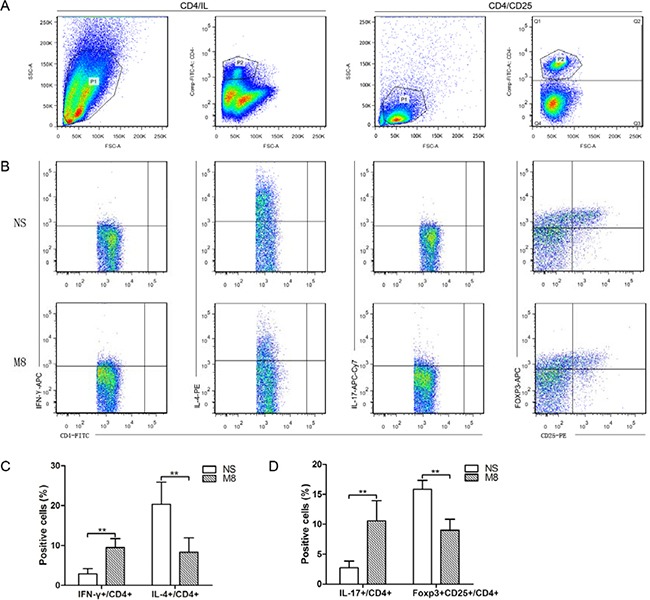
(**A**) Representative pictures for CD4+ T cells and CD4+CD25+ T cells. (**B**) Representative pictures for Th1 (a), Th2 (b), and Th17 (c), CD4+CD25+Foxp3+ Tregs (d). (**C**) The results of statistical analysis for the average percentages of Th1 and Th2 cells in different groups. (**D**) The statistical results for the average percentages of Th17 and CD4+CD25+Foxp3+ Tregs were determined by analyzing the FACS data. **P < 0.01.

Huber et al. demonstrated that Th2 cells or a switch from Th1 to Th2 may protect against atherosclerosis by limiting the Th1 cell response [12]. Recent studies revealed that IL-17 played an important role in atherogenesis and CD4^+^CD25^+^Foxp3^+^ Tregs cells have atheroprotective effects [3, 6, 7, 13]. We therefore examined CD4^+^ T-cells subtypes in the spleen after 24 weeks NS or METH treatment (Figure 3A and 3B). Our results revealed significantly increased Th1 and Th17 cells (9.47 ± 2.25 vs. 2.88 ± 1.29%, *P <* 0.01; 10.55 ± 3.41 vs. 2.73 ± 1.11%, *P <* 0.01; Figure 3C and 3D), and significantly reduced Th2 cells and CD4^+^CD25^+^Foxp3^+^ Tregs cells (8.31 ± 3.61 vs. 20.34 ± 5.54%, *P <* 0.01; 9.0 ± 1.83 vs. 15.86 ± 1.47%, *P <* 0.01; Figure 3C and 3D) in M8 group compared to NS group.

### Methamphetamine up-regulated systemic inflammation responses

**Figure 4:**
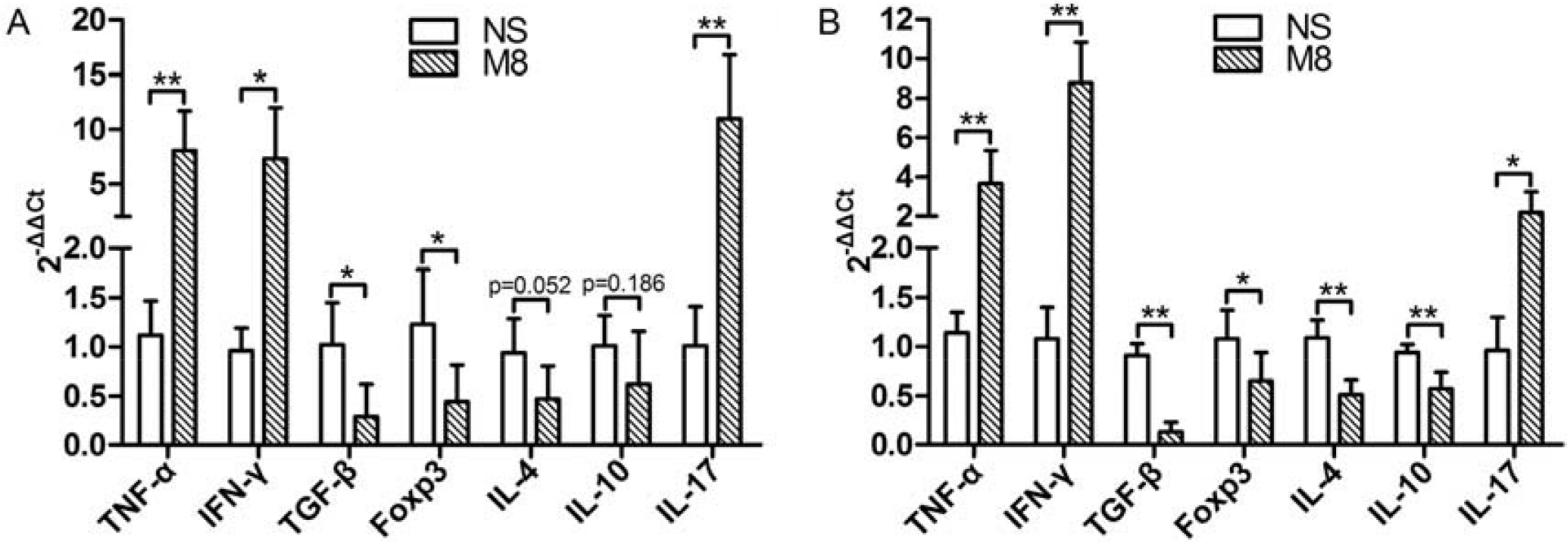
(**A**) Represent the mRNA levels of TNF-α, IFN-γ, TGF-β, Foxp3, IL-4, IL-10, and IL-17 in carotid artery. (**B**) Represent the mRNA levels of TNF-α, IFN-γ, TGF-β, Foxp3, IL-4, IL-10, and IL-17 in spleen. Values are presented as the mean ± SD. *P < 0.05, **P < 0.01.

We tested the mRNA levels for Th1 (IFN-γ) and Th2 (IL-4) in carotid artery (Figure 4A) and spleen (Figure 4B). Results demonstrated that IFN-γ expression was significantly up-regulated, but IL-4 expression was significantly down-regulated in M8 group compared to NS group (Figure 4A and 4B). IL-17 was significantly higher while Foxp3 was significantly lower in M8 group than in NS group (Figure 4A and 4B). Methamphetamine also significantly down-regulated the expressions of anti-inflammatory factors (TGF-β and IL-10, Figure 4A and 4B).

## DISCUSSION

The major finding of the present study is as follows: 1) Chronic METH treatment aggravated plasma dyslipidemia and atherosclerotic plaque formation in APOE−/− mice fed high cholesterol diet. 2) Chronic METH treatment induced the imbalance of Th1/Th2, and Th17/CD4^+^CD25^+^Foxp3^+^ Tregs in the spleen. 3) METH up-regulated the expression of proinflammatory IFN-γ TNF-αIL-17 and down-regulated the expression of anti-inflammatory IL-4, IL-10, TGF-β, and Foxp3 in carotid artery and spleen.

### METH treatment enhanced plasma dyslipidemia and plaque formation in APOE−/− mice fed a high-cholesterol diet

In our previous study, we showed that chronic METH treatment promoted atherosclerosis formation in APOE−/− mice fed normal chow [14]. However, the atherosclerotic plaque was not visible in APOE−/− mice fed normal chow [14], thus it is difficult to evidence the effects of chronic METH treatment on atherosclerotic plaque progression. In this study, chronic METH treatment was applied to APOE−/− mice fed high cholesterol diet. The presence of visible atherosclerotic plaque in this model makes it possible to validate the exact role of chronic METH treatment on plaque progression and our results showed that METH significantly enhanced atherosclerotic plaque formation in this model. To our surprise, present study demonstrated that chronic METH use also aggravated plasma dyslipidemia, although the underlying mechanism for the aggravated dyslipidemia remains elusive, this phenomenon might at least partly contribute to the observed aggravated atherosclerosis formation in this model. Further studies are needed to explore the exact mechanisms for the aggravated dyslipidemia post chronic METH application and to which extent this enhanced dyslipidemia might have contributed to the observed aggravated atherosclerotic plaque formation in this model.

### Role of the CD4+ cells balance on METH-enhanced atherosclerosis

Atherosclerosis is a multifactorial arterial wall disease, and immune and inflammation responses play major roles in the pathogenesis of atherosclerosis. It is known that T cells mediated chronic inflammation serves as a major player in atherogenesis [1]. Harms et al. demonstrated that METH treatment could induce changes of CD4 and CD8 T cell subsets and result in immunosuppression in a murine model [15]. Our results indicate that METH might enhance the progress of atherosclerosis by affecting the changes of CD4^+^ T cells subsets in the high cholesterol diet circumference in APOE−/− mice. Through the analysis of numbers of CD4^+^ T cells in spleen, we evidenced significantly increased proinflammatory Th1 and Th17 cell components and decreased anti-inflammatory Th2 and CD4+CD25+Foxp3+ Tregs cell components. The Th1/Th2 imbalance was a known stimulant for atherosclerosis development [5]. Previous study showed that Th2 cells or a switch from Th1 to Th2 may protect against atherosclerosis by limiting the Th1 cell response [16]. In line with above finding, our results showed that chronic METH could induce the transformation of CD4^+^ T cells to Th1 cells, this finding might thus be responsible for the observed enhanced atherosclerosis in this model. Th17 cells, a subset of CD4 cells capable of secreting proinflammatory IL-17, IL-6, and TNF-α cytokines, also play a crucial role on atherogenic pathogenesis [7, 13]. In contrast, TGF-β, which is produced by many cells, such as CD4^+^CD25^+^Foxp3^+^ Tregs, is a protective effector in atherosclerosis [3, 4]. In our studies, we evidenced an increase of Th17 cells and a decrease of CD4^+^CD25^+^Foxp3^+^ Tregs in spleen. Taken together, the increased ratio of Th1/Th2 and Th17/CD4^+^CD25^+^Foxp3^+^ Tregs post chronic METH might also be responsible for the observed enhanced formation of atherosclerotic plaque in this model. In this study, we found METH could increase plasma cholesterol level, which was accompanied by the up-regulation of Th17 cells and down-regulation of Tregs. These results suggest that METH-induced lipid disorder might result in the imbalance of Th17/Tregs and this finding is consistent with results from previous studies [17–19].

### Methamphetamine and inflammation

Results from previous studies and our present study found that MTETH could alter the production of both inflammation and anti-inflammation components [1, 20, 21]. In animal experiments, both high and low dose METH was shown to induce proinflammatory cytokines production in brain [22–25]. We detected inflammation-related mRNA expression levels of cytokines in the carotid artery and spleen and found that the mRNA expressions of proinflammatory cytokines including TNF-α, IFN-γ, and IL-17 were significantly up-regulated in these tissues post chronic METH treatment, while the mRNA expression levels of anti-inflammatory cytokines including TGF-β, Foxp3, IL-4, and, IL-10 [26] were significantly down-regulated in these tissues post chronic METH treatment. Thus, chronic METH induced evident imbalance in favor of proinflammatory status in these tissues. It is thus reasonable to speculate that the enhanced atherosclerosis formation post chronic administration of METH in this model might also be mediated through the inhibition of immune cells secreting the anti-inflammatory cytokines and through the activation of immune cells secreting inflammatory cytokines.

### Study limitation

The fact that chronic METH use enhanced the atherosclerotic plaque formation in ApoE−/− mice fed a high cholesterol diet might mean that the plaque stability might also be affected by METH on top of high cholesterol diet in this model and induce more vulnerable plaque, however, present study did not focus on this issue, future studies are warranted to explore the plaque stability status in METH treated ApoE−/− mice fed a high cholesterol diet and the impact of other drugs, which have plaque stabilization capacities, on plaque vulnerability in this model. Moreover, besides the potential mechanistic observed in this study, other inflammatory factors, such as macrophage 1 and 2, galectin 3 and 1 may be involved in the METH-induced atherosclerotic plaque formation in this model, future studies with examinations on above inflammatory factors might help to reveal the potential mechanisms of atherosclerosis formation post METH use in this model. It is to note that present study results are based on data derived form a small animal number, and one dose: 8 mg/kg/day, and this dose was chosen based on our previous study showing this dose promoted atherosclerosis formation in ApoE−/− mice fed normal diet [14], and studied just on one time point (24 weeks), these factors indicate the preliminary nature of reported study results. Future studies with variable dose, multiple study time points and with more animal number in each study group are essential to validate the results reported from present study.

In conclusion, our results indicate that chronic METH could aggravate atherosclerosis formation in APOE−/− mice fed a high cholesterol diet through inducing immune imbalance in favor of the proinflammatory status. Together with the aggravated plasma dyslipidemia, immune imbalance in favor of the proinflammatory status could be one of the mechanisms of enhanced atherosclerosis observed in this model. Further studies are warranted to test if therapy options to re-establish the immune balance in favor of anti-inflammatory status as a novel therapy strategy to attenuating/retarding the atherosclerosis progression.

## MATERIALS AND METHODS

### Animals and diets

Six-week-old male ApoE−/− mice with the C57BL/6J background were obtained from Beijing HFK Bioscience Co. (China) and were kept in specific pathogen-free facility. METH was dissolved in saline and the final concentrations were 0.8 mg/ml. Animals were randomly divided into saline control (NS, *n* = 5) and METH group (8 mg/kg/day through intraperitoneal injection, M8, *n* = 6) and treated for 24 weeks. During NS or METH treatment, mice were fed with a high-cholesterol diet containing 1.25% cholesterol and 20% lard. The mice were housed under conditions of controlled illumination (12:12-h light/dark cycle), humidity and temperature. They were allowed ad libitum access to water. All experimental procedures were performed under the guidelines of the Care and Use of Laboratory Animals (Science and Technology Department of Hubei Province, China, 2005) and approved by the Institutional Animal Research and Ethics Committee of Huazhong University of Science and Technology. All experiments were conducted in compliance with the ARRIVE guidelines and in accordance with the National Institutes of Health guide for the care and use of Laboratory animals (NIH Publications No. 8023, revised 1978).

### Blood and tissue samples collection and storage

The mice were deep anesthetized under intraperitoneal pentobarbital (1%, 40 mg/kg) and blood was collected through abdominal vena cava at 24 weeks after intervention. Plasma was collected after the centrifugation and was stored at –80°C until use. Thereafter, mice were sacrificed by cervical dislocation, spleen were removed and stored at –80°C for biochemistry analysis.

### Atherosclerotic plaque assessment

After *in situ* perfusion with cold PBS and subsequently with cold buffered formalin, the arch and thoracic portion of the dorsal aorta were dissected free from the thoracic cavity and heart. The entire aorta was isolated from the arch to the aortic-iliac bifurcation. After removing the adventitia and adipose tissue it was placed in 10% neutral buffered formalin overnight. The aorta was then opened lengthwise, and pinned flat in a wax bottomed dissecting pan. The tissue was stained for 15 minutes with 0.5% Oil Red O in acetone and 70% ethanol (1:1). The tissue was decolorized for 5 minutes using 80% ethanol, and then washed gently with water for several minutes. The en face preparations were digitally photographed and then quantified using Optimas 6.5 software, and percent of plaque coverage was calculated.

### Immunohistochemistry

For the analysis of atherosclerotic plaque composition, the heart was sectioned parallel to the atria and 5˜7-μm sections starting from the three valve cusps of the aortic sinus were fixed in 4% formaldehyde, processed, and embedded in OTC compound. Then staining was performed with the following antibodies: purified anti-F4/80 Ab (1:600) for monocyte and macrophages, purified anti-smooth muscle actin (α-SMA) Ab (1:200) for SMCs. Anti-F4/80 (MCA497GA) was purchased from Bio-Rad (USA), anti-SMA (BM0002) was purchased from Boster (China). The purified antigen-antibodies were detected by biotinylated secondary antibodies in combination with streptavidin biotin complex and DAB system. The images of stained aorta were captured by digital camera, and the results were analyzed using Optimas 6.5 software.

### Real-time polymerase chain reaction

Total RNA was extracted from carotid aorta and spleen using Total RNA kit (Takara, Japan) according to the manufacturer's instructions. Reverse transcription and cDNA synthesis were accomplished using RNA PCR kit (Takara, Japan). Real-time polymerase chain reaction was performed by Step One SYBR Green Mix Kit (Takara, Japan) and ABI Prism Sequence Detection System (Applied Biosystems, USA) according to the manufacturer's instructions. The conditions of amplification reaction were 95°C for 30 sec, 95°C for 5 sec, 60°C for 30 sec, and PCR was done for 40 cycles. Relative PCR primers are shown in Table 2.

**Table 2:** RT-PCR forward/reverse (F/R) primers sequences

Forward/Reverse	Sequence (5′–3′)
IL-17	F: 5′-ATGTGGTGGTCCAGCTTTCC-3′ R: 5′-CTCAGACTACCTCAACCGTTCC-3′
IL-10	F: 5′-ACCTGCTCCACTGCCTTGC-3′ R: 5′-GGTTGCCAAGCCTTATCGG-3′
IL-4	F: 5′-CACTCTCTGTGGTGTTCTTCGTT-3′ R: 5′-GTCATCCTGCTCTTCTTTCTCG-3′
FOXP3	F: 5′-GCAGGGATTGGAGCACTTGTT-3′ R: 5′-TTGGTTTACTCGCATGTTCGC-3′
TGF-β	F: 5′-GCTTGCGACCCACGTAGTAGA-3′ R: 5′-AGCCCTGGATACCAACTATTGC-3′
TNF-α	F: 5′-CCTACCTTCAGACCTTTCCAGAT-3′ R: 5′-GGCCTTCCAAATAAATACATTCA-3′
IFN-γ	F: 5′-GTCTCTTCTTGGATATCTGGAGGA-3′ R: 5′-ATTCAATGACGCTTATGTTGTTGC-3′
β-Actin	F:5′-TGAGAGGGAAATCGTGCGTGAC-3′ R:5′-GCTCGTTGCCAATAGTGATGACC-3′

### Flow cytometry

Twenty-four weeks after NS or METH treatment, the lymphocytes from the spleen were isolated with Ficoll-Paque Plus method, erythrocytes were removed by RCLB. Fluorescent staining of CD4^+^CD25^+^Foxp3^+^ Tregs, Th1, Th2, and Th17 cells was performed with the following antibodies obtained from eBioscience (CA, USA): Anti-CD4-FITC Ab, Anti-CD25-PE Ab, Anti-IL-17-APC Ab, Anti-IL-4-PE Ab, Anti-IFN-γ-APC Ab, and Anti-Foxp3-APC Ab.

For the detection of CD4+CD25+Foxp3^+^ Tregs, lymphocytes from the spleen were stained with anti-CD4- FITC and anti-CD25-PE, and then stained with anti-Foxp3-APC after fixation and permeabilization according to the manufacturer's instructions. For analysis of Th1 (CD4+IFN-γ+), Th2 (CD4^+^IL-4^+^), and Th17 (CD4^+^IL-17^+^), the lymphocytes were suspended at a density of 2 × 10^6^ cells/mL in complete culture medium. The cell suspension (1 ml) was transferred to each well of 24-well plate. Cultures were stimulated with phorbol myristate acetate (PMA, 20 ng/mL) plus ionomycin (1 μg/mL) (Alexis Biochemicals, San Diego, CA) for 4 h in the presence of 2 μmol/ml monensin (Alexis Biochemicals). The incubator was set at 37°C under a 5% CO_2_ environment. After 4 h of culture, lymphocytes were collected for staining according to the instructions. Fixation and permeabilization were reached before staining with IFN-γ, IL-4, or IL-17 antibody. Flow cytometric acquisition was performed using a FACS Calibur (BD Immunocytometry Systems, USA) and all analysis was performed using Flowjo software (Treestar Inc., OR, USA).

### Weight monitoring and plasma lipids measurements

Body weight was recorded at baseline and every two weeks after the first administration of NS or METH. The plasma obtained previously was used to detect lipids levels of plasma total cholesterol (TC) and triglyceride (TG) using commercial kits from DIASYS company, Shanghai. High density lipoprotein cholesterol (HDL-C) and low-density lipoprotein (LDL-C) were measured by using commercial kits from SEKISUI company, Japan917).

### Statistical analysis

Data were showed as mean ± SD. A student's *t* test was used to analyze the statistical difference between NS and METH groups and A *p* value < 0.05 was considered as statistically significant.

## Abbreviations

ApoE: Apolipoprotein E
α-SMA: α-smooth muscle actin
Foxp3: transcription factor forkhead box P3
TNF-α: tumor necrosis factor-α
IFN-γ: interferon-γ
TGF-β: transforming growth factor-β
Th1, 2, and17: T-helper 1, 2, and17
